# A Mobile App for Longterm Monitoring of Narcolepsy Symptoms: Design, Development, and Evaluation

**DOI:** 10.2196/14939

**Published:** 2020-01-07

**Authors:** Laury Quaedackers, Jan De Wit, Sigrid Pillen, Merel Van Gilst, Nikolaos Batalas, Gert Jan Lammers, Panos Markopoulos, Sebastiaan Overeem

**Affiliations:** 1 Center for Sleep Medicine Kempenhaeghe Heeze Netherlands; 2 Department of Industrial Design Eindhoven University of Technology Eindhoven Netherlands; 3 Department of Communication and Cognition, Tilburg Center for Cognition and Communication Tilburg University Tilburg Netherlands; 4 Department of Electrical Engineering Eindhoven University of Technology Eindhoven Netherlands; 5 Sleep-Wake Center SEIN Heemstede Netherlands; 6 Department of Neurology Leiden University Medical Center Leiden Netherlands

**Keywords:** outcome measure, hypersomnia, patient-related outcome measure, PROM, mHealth, symptom monitoring

## Abstract

**Background:**

Narcolepsy is a chronic sleep disorder with a broad variety of symptoms. Although narcolepsy is primarily characterized by excessive daytime sleepiness and cataplexy (loss of muscle control triggered by emotions), patients may suffer from hypnagogic hallucinations, sleep paralysis, and fragmented night sleep. However, the spectrum of narcolepsy also includes symptoms not related to sleep, such as cognitive or psychiatric problems. Symptoms vary greatly among patients and day-to-day variance can be considerable. Available narcolepsy questionnaires do not cover the whole symptom spectrum and may not capture symptom variability. Therefore, there is a clinical need for tools to monitor narcolepsy symptoms over time to evaluate their burden and the effect of treatment.

**Objective:**

This study aimed to describe the design, development, implementation, and evaluation of the Narcolepsy Monitor, a companion app for long-term symptom monitoring in narcolepsy patients.

**Methods:**

After several iterations during which content, interaction design, data management, and security were critically evaluated, a complete version of the app was built. The Narcolepsy Monitor allows patients to report a broad spectrum of experienced symptoms and rate their severity based on the level of burden that each symptom imposes. The app emphasizes the reporting of changes in relative severity of the symptoms. A total of 7 patients with narcolepsy were recruited and asked to use the app for 30 days. Evaluation was done by using in-depth interviews and user experience questionnaire.

**Results:**

We designed and developed a final version of the Narcolepsy Monitor after which user evaluation took place. Patients used the app on an average of 45.3 (SD 19.2) days. The app was opened on 35% of those days. Daytime sleepiness was the most dynamic symptom, with a mean number of changes of 5.5 (SD 3.7) per month, in contrast to feelings of anxiety or panic, which was only moved 0.3 (SD 0.7) times per month. Mean symptom scores were highest for daytime sleepiness (1.8 [SD 1.0]), followed by lack of energy (1.6 [SD 1.4]) and often awake at night (1.5 [SD 1.0]). The personal in-depth interviews revealed 3 major themes: (1) reasons to use, (2) usability, and (3) features. Overall, patients appreciated the concept of ranking symptoms on subjective burden and found the app easy to use.

**Conclusions:**

The Narcolepsy Monitor appears to be a helpful tool to gain more insight into the individual burden of narcolepsy symptoms over time and may serve as a patient-reported outcome measure for this debilitating disorder.

## Introduction

### Background

Narcolepsy is a chronic neurological sleep disorder caused by a deficiency of the hypothalamic neurotransmitter, hypocretin [1,2]. Patients with narcolepsy experience a broad range of symptoms, the most common being excessive daytime sleepiness, manifested not only as attacks of falling asleep at inappropriate times but also as difficulties with concentration and memory [3-5]. Patients often experience attacks of muscle weakness triggered by emotions, called cataplexy [6]. These symptoms, together with hypnagogic hallucinations, sleep paralysis, and disturbed nocturnal sleep, are referred to as the classic pentad of narcolepsy [7]. However, the spectrum of narcolepsy symptoms is more extensive, including several symptoms that are not directly related to sleep. Increased weight and the presence of psychiatric symptoms (such as affective disorders, eating disorders, attention-deficit hyperactivity disorder, and schizophrenia) have been described in several studies [8-13]. Social functioning seems to be impaired in children with narcolepsy, and quality of life is negatively affected [14,15]. The severity of the symptoms varies greatly among patients and there also is significant day-to-day variance within patients. Differences in experienced severity are influenced by medication effect and medication tolerance as well as circumstances of daily living and individual variation in coping strategies. Both the broad symptom spectrum and symptom variability stress the need for individually tailored care in narcolepsy.

Narcolepsy affects approximately 1 in 2000 people but is often not correctly diagnosed. Delayed recognition is a well-described clinical problem and patients often receive a diagnosis several years after the onset of symptoms [16]. The core symptoms of excessive daytime sleepiness and cataplexy are essential in the diagnostic process, but quality of life of narcolepsy patients is not determined by these 2 symptoms alone. Maski et al [17] reported that patients stated their most troublesome symptoms were general fatigue and subjective cognitive complaints. Moreover, there are doubts about the meaning of the core symptoms in relation to the subjective severity of narcolepsy. For example, only a moderate correlation was found between the Epworth Sleepiness Scale and the results of the Multiple Sleep Latency Test, the current objective standard to assess sleepiness [18].

In a recent review by Kallweit et al [19], 7 narcolepsy questionnaires were evaluated. None of these questionnaires seem to fulfill the requirements to function as a tool to monitor the extensive spectrum of narcolepsy symptoms over a longer period. Digital solutions in health care (mobile health) may provide solutions in this area. With the development of an app designed to run on a mobile device, new possibilities emerge to record long-term subjective data. Digital data collection is perceived as less invasive and time consuming, which can have a positive effect on long-term adherence. Moreover, instead of the frequency of symptoms, which is used as a key marker of severity in all questionnaires, probing the *impact* of symptoms might help to understand the narcolepsy patient better and lead to more personalized treatment choices.

### Objectives

Here, we describe the design, development, and evaluation of the Narcolepsy Monitor, a companion app for long-term symptom monitoring in narcolepsy patients. The purpose of the app is to allow patients to frequently self-report on the subjective burden of a variety of narcolepsy symptoms over time. During development, we aimed to make the app personalized, minimally invasive, and able to provide data meaningful for both patients and caregivers. We used a theory-driven and user-driven iterative approach, where user feedback supported content composition and ensured usability. We then evaluated the Narcolepsy Monitor with regard to usability and user experience feedback.

## Methods

**Figure 1:**
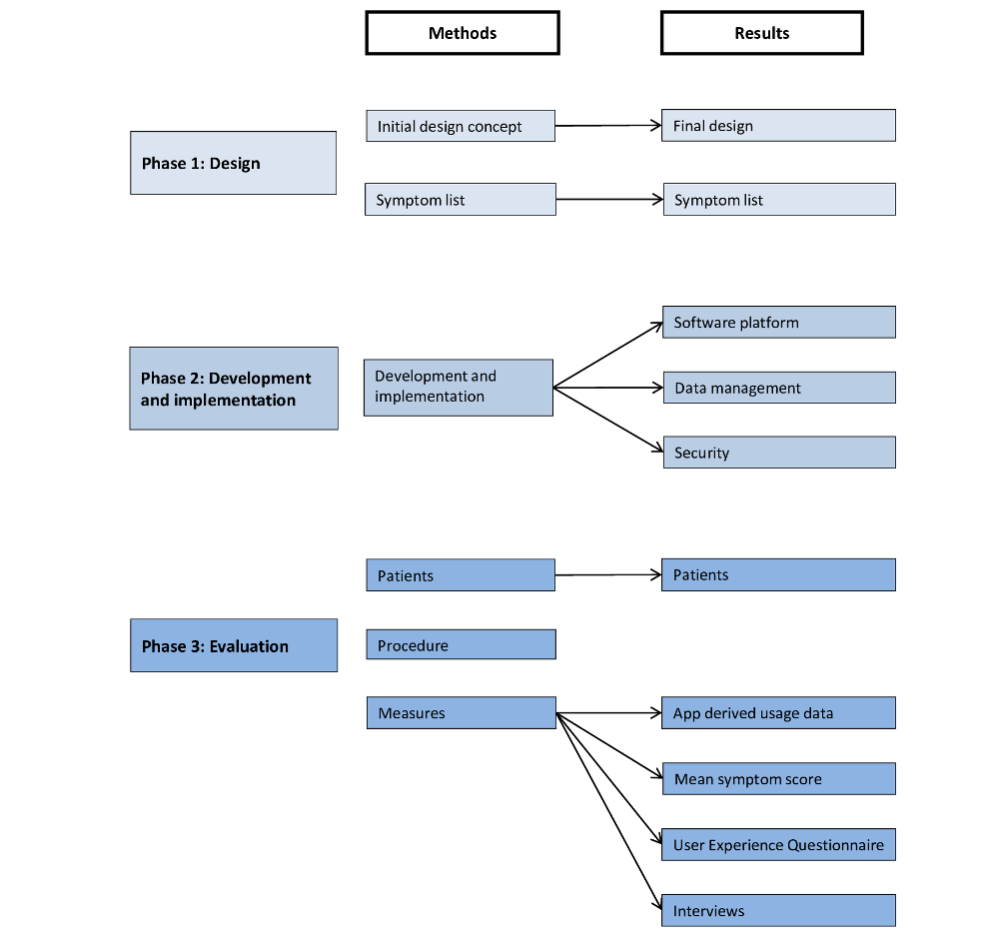
The different phases of the development of the Narcolepsy Monitor.

The development process of the Narcolepsy Monitor comprised 3 phases: (1) design, (2) development and implementation, and (3) evaluation (Figure 1).

### Phase 1: Design

#### Initial Design Concept

**Figure 2:**
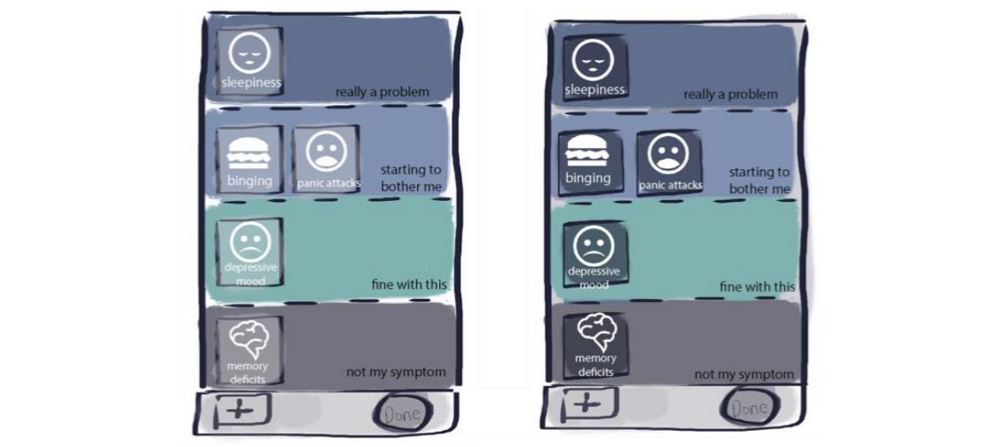
Concept for a symptom ranking screen.

Under the supervision of the authors, a group of 5 students involved in the User System Interaction Program of the Eindhoven University of Technology (TU/e), started in 2016 with an exploration on the design statement, “Design a mobile app for narcolepsy patients that runs on their personal device, to self-monitor the subjective severity of narcolepsy symptoms.” A total of 4 high-level requirements were formulated that would be informative for the design: (1) efficient and easy to use, (2) personalized, (3) highly visual, and (4) long-term commitment. After several iterations, the group reached consensus on the concept of a *ranking screen*, where all relevant symptoms would be visible in 1 overview (Figure 2). This concept was further refined taking all the requirements into account.

#### Symptom List

The initial domains to be covered by the Narcolepsy Monitor were defined based on a literature review and expert opinion. When defining the symptom list, there was a specific intention to cover the large variation in complaints expressed by narcolepsy patients. Several preliminary versions of the symptom list were developed and reviewed by 3 physicians and 3 patients.

### Phase 2: Development and Implementation

During the development of the Narcolepsy Monitor, special attention was given to data management and security aspects. The first version of the requirements with regard to data management, safety, and privacy was reviewed by the local medical ethics committee, which led to suggestions for further improvement. Consultation with a hospital information technology specialist led to a further augmentation of the security structure.

### Phase 3: Evaluation

#### Patients

Patients were recruited from January 2017 till August 2017, from the outpatient clinics of Sleep Medicine Center Kempenhaeghe (Heeze, NL) and Sleep-Wake Center SEIN (Heemstede, NL). Patients were aged 18 years or above and included if they fulfilled the criteria of narcolepsy type 1 or 2 according to the standards of the International Classification of Sleep Disorders 3: Diagnostic and Coding Manual [20]. Patients were excluded if they were not able to read or speak Dutch or had severe cognitive impairments, comorbid medical diagnosis or psychiatric illness.

#### Study Procedure

Patients with a diagnosis of narcolepsy were informed by their treating physician about the study, both orally and in writing. After the patient agreed to participate in the study, written consent forms were signed. A manual on how to download and install the Narcolepsy Monitor was sent to the home address of the participants with the request to start using the app. Patients were asked to follow the instructions of the app for 30 days. Thereafter, the patients were asked to fill in the user experience questionnaire (UEQ; see User Experience Questionnaire section) [21]. In addition, patients were also invited for a personal in-depth interview through a secure consultation on the Web conducted by the first author. The medical ethics committee of the Maxima Medical Center in Veldhoven, NL (METC number: N16.084) approved this study.

#### Measures

#### Mean Symptom Score

Symptom severity was expressed as the mean score per symptom. Categories were given a numeric value (*not relevant*=0, *not really a problem*=1, *a little problem*=2, and *really a problem*=3). Although patients did not rate their symptoms on a daily basis, we assumed that the rating remained the same until a patient moved the icon to another category. As a result, for each day, a value was used in the calculations. The sum of these values was divided by the number of days the app was used in total to obtain an average score for the respective symptom (Table 1).

**Table 1:** Mean symptom scores and dynamics of the symptoms.

Symptoms in the app	Patients that chose this symptom, n	Severity score (N=7), mean (SD)	Number of changes, mean (SD)
Daytime sleepiness	7	1.8 (1.0)	5.6 (3.7)
Lack of energy	7	1.6 (1.4)	3.4 (2.4)
Often awake at night	7	1.5 (1.0)	3.6 (4.4)
Difficulty achieving things	6	1.1 (1.0)	2.2 (2.0)
Difficulty concentrating	7	1.1 (0.9)	3.1 (1.5)
Increase in weight	5	0.9 (1.1)	2.0 (2.4)
Difficulty with memory	4	0.8 (1.0)	1.0 (1.3)
Binge eating	5	0.7 (0.8)	1.9 (1.6)
Problems at work	4	0.6 (0.7)	1.6 (2.1)
Agitation	5	0.6 (0.8)	1.4 (1.3)
Cataplexy	5	0.6 (0.9)	2.3 (1.8)
Lifelike dreams	2	0.5 (0.9)	0.5 (1.2)
Problems with relationships	4	0.5 (0.8)	1.1 (2.0)
Sleep paralysis	2	0.4 (0.8)	0.4 (1.0)
Problems with libido	5	0.4 (0.7)	0.8 (0.9)
Sadness	2	0.3 (0.5)	0.7 (1.7)
Feelings of anxiety or panic	2	0.1 (0.2)	0.3 (0.7)
Problems at school	0	0.0 (0.0)	0.0 (0.0)

#### App-Derived Usage Data

The following variables were automatically retrieved from the app for analysis: (1) period during which the app was used, (2) number of days during which the app was opened, (3) number of different symptoms ranked, (4) total number of changes in all symptoms, and (5) number of changes per symptom (Table 2).

**Table 2:** App-derived usage data.

Usage data variables	Minimum	Maximum	Mean (SD)
Period for which app was used (days)	18	73	45.3 (19.2)
Percentage of days the app was opened	13.2	57.6	35 (16.3)
Number of symptoms ranked	9	16	11.7 (3.1)
Total number of changes made (per month)	14.2	48.3	31.6 (13.3)

#### User Experience Questionnaire

The UEQ is an easy-to-apply, reliable, and valid measure to assess user experience of interactive products [21]. The UEQ comprises both classical usability aspects (efficiency, perspicuity, and dependability) and user experience aspects (originality and stimulation). The scales of the UEQ can be grouped into pragmatic quality (perspicuity, efficiency, and dependability) and hedonic quality (stimulation and novelty). Pragmatic quality describes task-related quality aspects; hedonic quality describes the nontask-related quality aspects. Scale values above +1 indicate a positive impression of the users concerning this scale and values below −1 indicate a negative impression.

#### Interviews

Interviews varied in length, with a maximum of 66 min, and started with the open question: “How did you experience the use of the narcolepsy app?” This was followed by probing questions, further investigating the subjective experiences with the Narcolepsy Monitor. At the end, the interviewer checked the topic list to see if all subjects had been covered. Inclusion of patients stopped after reaching data saturation. All audio recordings were transcribed, and qualitative thematic analysis was applied. Recorded interviews were transcribed verbatim by a company specialized in transcribing for scientific purposes. The first author started reading the interviews several times to become acquainted with them. Afterward, the transcribed text was copied into Microsoft Excel, where each part of the text fragment was labeled with initial open codes after which several coding iterations were performed. Codes with similar content were grouped into themes. To support this process and enhance understanding of the results, 3 authors (LQ, SO, and SP) made mind maps, searching for themes and connection among themes.

#### Data Analysis

Data analysis was performed with SPSS, version 25, by using descriptive statistics. Data are shown as mean (SD) unless otherwise specified.

## Results

### Phase 1: Design

#### Final Design

**Figure 3:**
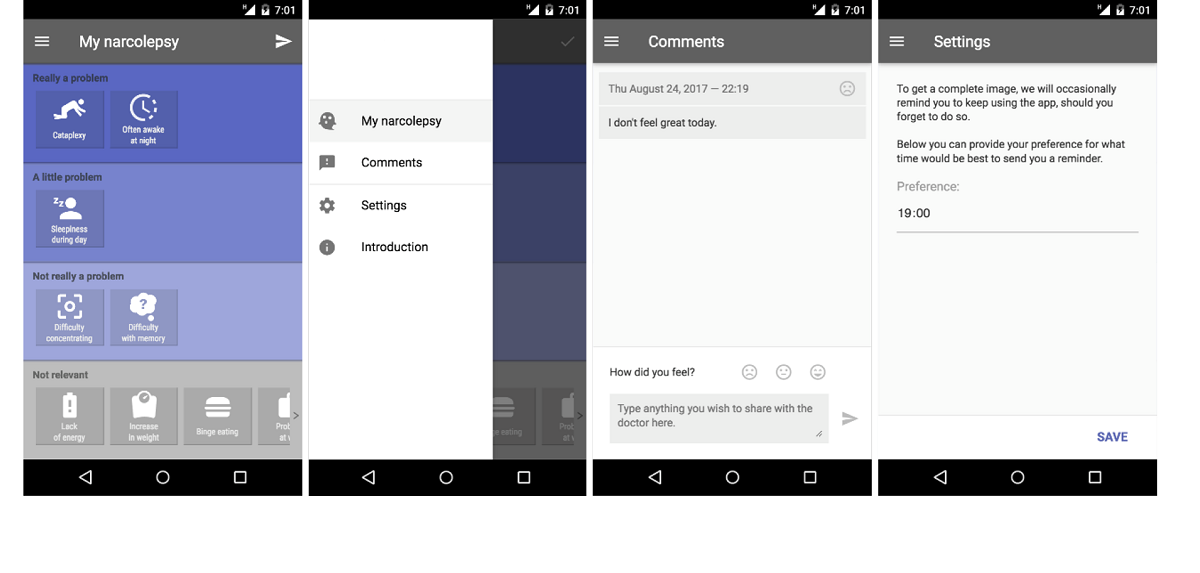
Final design of the Narcolepsy Monitor. The first panel shows the ranking environment in which patients list the narcolepsy symptom according to the subjectively experienced severity. The second panel shows the menu in which one can choose to navigate to the ranking screen, the comment section, personal settings and the introduction. In the third panel the possibility to add comments is shown. The last panel shows the possibility to adjust the time for the reminder to use the app.

We decided upon the core interaction concept in which a symptom ranking screen with all relevant symptoms is visible in 1 overview. Symptoms are represented as icons that can be dragged and dropped to zones of varying severity and changed if their severity changes. Importantly, severity is rated not by the *frequency* of a symptom but by the *burden* it poses to the individual. Patients can choose between *not really a problem*, *a little problem*, and *really a problem*. The advantage of this approach is that subjective symptom severity is indicated on a relative scale with other symptoms present in the same overview. This scaling also *normalizes* symptoms toward each other, removing differences in, for example, a pure frequency of occurrence. Figure 3 shows the version of the Narcolepsy Monitor that was evaluated here.

#### Symptom List

**Figure 4:**
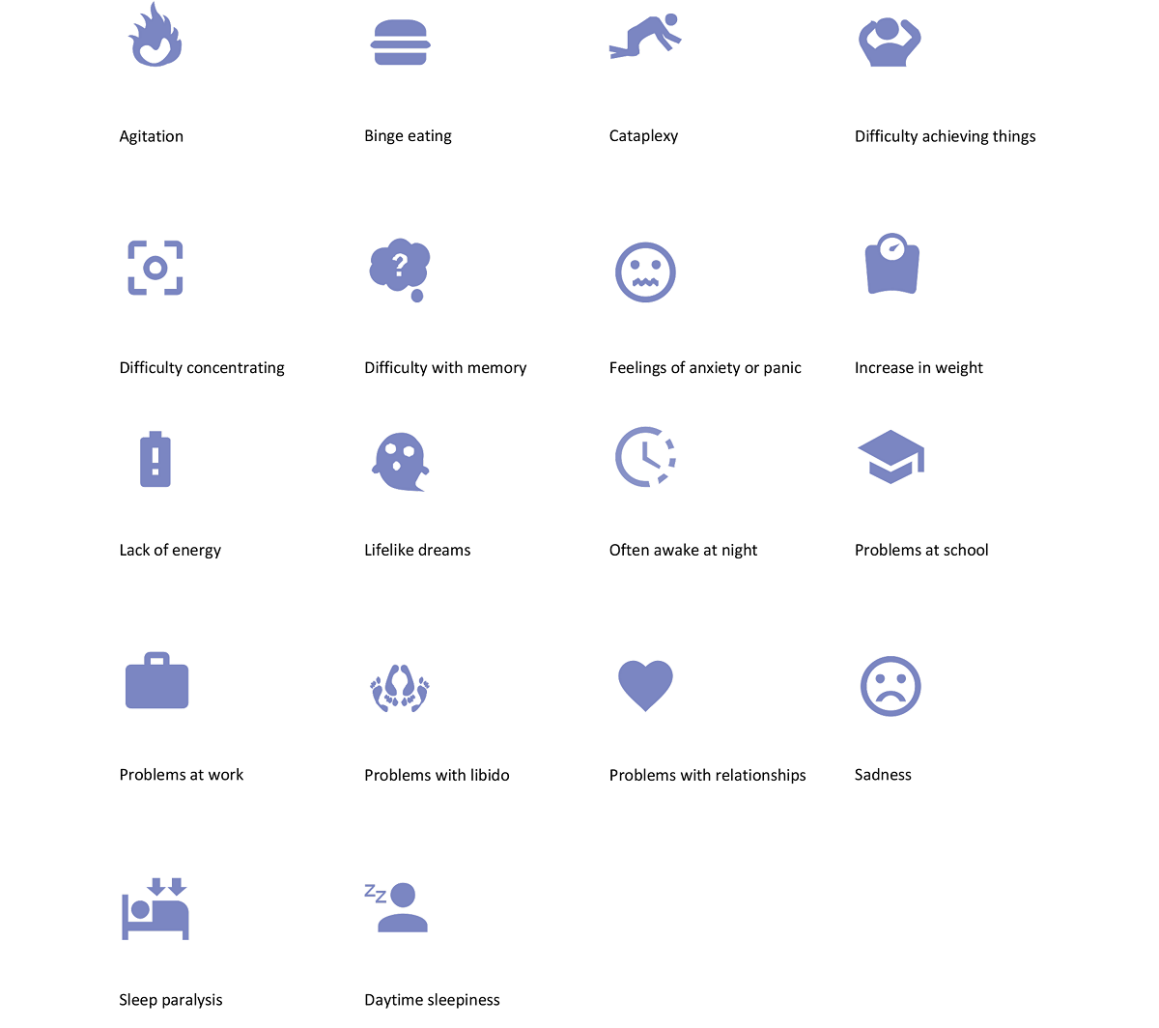
List of symptoms included in the Narcolepsy Monitor.

The final set of 18 symptoms that can be ranked is shown in Figure 4, together with their iconic representation in the app. These symptoms include the classical pentad of narcolepsy, augmented with other symptoms that are often experienced. These are supplemented by symptoms more indirectly related to the condition, including psychiatric and cognitive aspects. Overall, 3 patients were asked to give feedback on the icons used to represent the narcolepsy symptoms. Furthermore, patients were asked if any symptoms were missing and if there were inappropriate symptoms on the list.

### Phase 2: Development and Implementation

#### Software Platform

**Figure 5:**
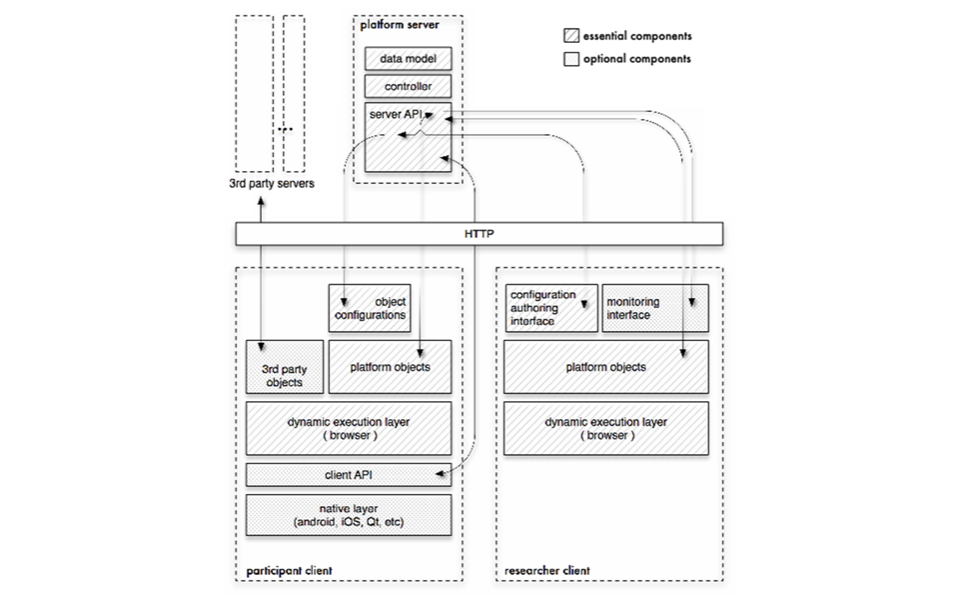
Tempest architecture.

The Tempest platform, designed by N Batalas and P Markopoulos, was used as the basis on which the narcolepsy app was implemented. The Tempest platform is programmable, modular, and extendable, and through extensive use has been validated as fitting the needs of practitioners of a diverse range of fields [22-24]. It comprises a Web app (see Figure 5; this is referred to as *researcher client*) that allows the researcher to compose the interfaces and to arrange the manner in which they are to be displayed in terms of sequence and conditional logic, akin to simple imperative programming. A client app to study participants (referred to as *participant client* in Figure 5) renders HTML and JavaScript-based user interfaces. The client app can be distributed as either a pure Web app or an Android app, which then allows for more native mobile phone functionality such as setting alarms. Finally, it also comprises a Web server or database component that stores and communicates configuration and data between participant and researcher clients.

#### Data Management

The server side of the Tempest framework is responsible for receiving the data of the participants and storing them in a central database. This server was managed by the TU/e.

#### Security

With regard to safety, a number of security measures were taken. This includes the security of the server, for example, by encrypting all communication channels and by implementing an update protocol for the operating system and the installed software package. As the most important security measure, personal information, such as the patients’ names, contact information, diagnoses, and details regarding treatment, were not stored anywhere on the server or on the patient’s device. All data that were logged through the system could only be linked to patients through a unique and randomly generated personal identification number and only the executive researcher (LQ) had access to a list that links this identification number to a patient.

### Phase 3: Evaluation

#### Patients

Overall, 10 patients were included for this pilot study; 3 patients dropped out. As a result, a total of 7 patients (females) participated in the pilot study, with a mean age of 44 (SD 11.5) years.

#### App-Derived Usage Data

Table 2 shows data regarding the use of the Narcolepsy Monitor. Although patients were asked to use the app for 30 days, the range of period of use varied from 18 to 73 days. On an average, the app was opened on 35% of the days during the study period. Patients, on average, rated 11.7 symptoms out of the 18 options. Per month, patients on average made a total of 31.6 changes in the ranking of symptoms (a change was defined as a single change in 1 symptom), including the first use ranking of the symptoms.

#### Mean Symptom Scores

**Figure 6:**
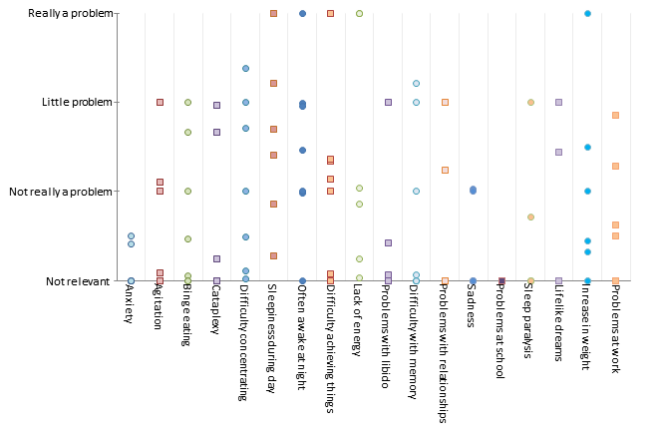
Mean score per patient per symptom. Patients provided input on all 18 symptoms. Bullet points may capture the input of more than 1 patient, especially on the “not relevant” and “really a problem” level.

In Table 1, the mean score per symptom for all 7 patients combined is listed. The table also gives insight into the dynamics of the symptoms. Daytime sleepiness, lack of energy, and often awake at night were the 3 highest scored symptoms. As nobody attended school, the participants did not rank school problems. The symptoms rated as least bothering were problems with libido, sadness, and feelings of anxiety and panic. Cataplexy only had the 11th position in the ranking of the level of subjective severity, with a mean score of 0.5 (SD 0.9), despite being regarded as one of the most defining symptoms of narcolepsy. Lifelike dreams was 12th, with a mean score of 0.5 (SD 0.9), and sleep paralysis, with a mean score of 0.4 (SD 0.7), took the 14th position. Note that the latter 3 (cataplexy, lifelike dreams, and sleep paralysis) are part of the classic pentad of narcolepsy but nevertheless scored relatively low. Figure 6 illustrates the variance in mean score over patients per symptom.

The symptoms daytime sleepiness, difficulty concentrating, often awake at night, and lack of energy were used by all patients. The symptom daytime sleepiness varied the most (5.6 [SD 3.7]). This was followed by often awake at night (3.6 [SD 4.6]) and lack of energy (3.4 [SD 2.4]). The levels of severity of lifelike dreams, sleep paralysis, and feelings of anxiety or panic were changed the least (0.5 [SD 1.2], 0.4 [SD 1.0], and 0.3 [SD 0.7], respectively).

#### User Experience Questionnaire

Perspicuity was rated very high (mean 2.1 [SD 0.3]), whereas novelty of the approach received the lowest rating (mean 0.3 [SD 0.8]). Pragmatic quality had a mean score of 1.4, followed by attractiveness with a score of 1.0 and hedonic quality with a score of 0.5.

#### Interviews

A total of 7 interviews took place after which inclusion stopped because of saturation of information. The average interview time was 36 min, varying between 15 min and 66 min. During the interviews, we identified 3 major themes: (1) reasons to use, (2) usability, and (3) features. Table 3 shows illustrative examples of patients’ quotes matching these themes.

**Table 3:** Examples of quotes matching the themes.

Theme and subtheme		Quote
**Reasons to use**		
	Personal value for patients	“Especially when one does not know what influences his or her complaints, the app could be a useful way to speed that process up.” (patient 7415)
	Type of setting	“I would be absolutely fine with using the app if my doctor would ask me to.” (patient 7376)
**Usability**		
	Ease of use	“It is simple to use, but not to simple. People should be able to understand it, just shifting around with the icons and then push the send button.” (patient 7058)
	Tutorial step	“The first time I started using the app, it was unclear to me that I had to make changes every day. So, after putting all my symptoms in the app, I started using the comments to give my input.” (patient 7368)
	Levels of subjective severity	“With three categories, there is little room to vary in complaints. Because regardless whether I have a good day or a bad day, my main complaint is still ‘really a problem.’” (patient 7415)
	Icons	“For me, the icons were clear and appropriate” (patient 5075)
	Procedure for sending information	“The thing that is a bit odd to me, is that is seems like that when you do not make any changes in the ranking, you cannot send the information. If I feel the same, I should not have to make any changes and be able to send it anyway.” (patient 7058)
**Features**		
	Symptoms	“I suffer from headaches, which is more a consequence of a bad night or insufficient naps. It is not mentioned in the app, probably because it is to specific and not narcolepsy related, but for me it would be a valuable addition.” (patient 6695)
	Comments	“I did feel the urgency to explain fluctuations in my symptoms. Not only for my own learning, but also because I think it will add value for the doctor.” (patient 7415)
	Visualization	“The fact that I am not able to look back at the information that I have put in the app is not a very motivating factor.” (patient 7058)
	Personalizing	“...a sort of diary that follows your story and starts asking personalized questions.” (patient 7415)

#### Theme 1: Reasons to Use

The first theme was *reasons to use* that incorporates 2 subthemes: type of setting and personal value for patients.

- Type of setting: Patients reported that the request of the treating physician would be enough to start and keep using the app. Participation in scientific research would be a good motivator as well. Patients rated the app as suitable for long-term use.
- Personal value for patients: Patients referred to the app as a helpful means to get a speedier insight about how narcolepsy affects an individual and, as a result, finding ways to deal with the condition. The level of personal value would be influenced by the disease phase and the day-to-day variance patients experienced. A possible motivator for patients would be to have insight about the course of their own symptoms in terms of a visualization of their recorded data.

#### Theme 2: Usability

The second important theme we identified was the usability of the app. This theme comprised 5 subthemes:

- Ease of use: Patients rated the Narcolepsy Monitor as being clear and user friendly and appreciated the layout of the app. All found that the use of the app was not time consuming. None of the patients experienced using the app as an emotional burden, although few participants saw some risk in being confronted with the disease on a frequent basis.
- Tutorial step: We chose an interactive way to support the user with pop-ups while performing simple tasks for the first time. However, the instructions were not sufficient for some patients, which led to some erroneous use of the app. Instead of ranking the symptoms, 1 patient used the comment section on a daily basis to elaborate on the symptoms.
- Levels of subjective severity: Patients had several remarks on the levels of severity. First, some patients had trouble with the terminology. The category not relevant and not really a problem were sometimes deemed too similar. A few patients proposed a category not existing to be used instead of not relevant. Second, the number of severity levels was perceived as too limited, insufficiently allowing to record nuances in complaints. Patients preferred more levels of severity to give additional insight into the dynamics of their symptoms.
- Icons: Patients were positive about the icons of the app, which they rated as pleasant and clear. In the bottom bar of the ranking screen, a horizontal menu is shown that features all symptoms marked as not relevant. As there are 18 symptoms, not all of them are visible at first glance and an action of the user is required to make all icons visible.
- Procedure for sending information: The procedure for sending input was not clear to all patients. Especially, it was not clear enough whether the entered information had been saved or not. Patients referred to the possibility of automatic behavior (one of the symptoms of narcolepsy), which is a state of reduced arousal during which semipurposeful actions can be performed without someone realizing it. Therefore, patients would be more prone to forget their actions and would depend more on active feedback. For this reason, a send button was built in the app. As patients were under the assumption that pushing the send button was necessary, they felt compelled to always make changes to save the data. It was not clear enough that the send button activates automatically when something in the ranking was changed.

#### Theme 3: Features

In terms of the content of the Narcolepsy Monitor, 4 aspects were deemed important:

- Symptoms: First, patients appreciated the extended list of symptoms. Some patients suggested to add the option of an additional symptom, a category other symptom namely: …, which would be very personal. Some patients were not sure if they had to register symptoms that they assumed as not narcolepsy related, such as headaches.
- Comments: Patients experienced the possibility of adding comments as positive. Adding extra information makes it easier to track certain patterns in symptoms with regard to potential influencing factors.
- Visualization: The fact that a personal visualization of entered data was lacking was mentioned as a limitation by all 7 patients. Not receiving any feedback might conflict with the goal to use the app over a longer period. Patients wanted to have insights about when the app was opened and obtain an overview of symptoms scores, medication use, and comments over a longer period.
- Personalizing: In response to possible improvements, patients mentioned the idea of making the Narcolepsy Monitor more personal with the possibility of receiving more personalized feedback. For example, screening the comments for specific topics and probing the user with questions regarding this topic. Another suggestion was to include a signaling function, in case the severity of narcolepsy symptoms worsened.

## Discussion

### Clinical Findings

Narcolepsy is a debilitating chronic sleep disorder with a broad symptom spectrum of highly variable severity. To aid in the follow-up and treatment of narcolepsy patients, the Narcolepsy Monitor was developed as a new way to monitor the severity of narcolepsy symptoms over a longer period. The results show that the Narcolepsy Monitor not only has potential as a follow-up tool for individual patients but may also be used in a research setting to gain more insight into the overall clinical picture of narcolepsy and the influence of individual symptoms on everyday life.

The ranking of symptoms based on the level of subjective severity confirms the notion of a broad symptom spectrum in narcolepsy exceeding the classical pentad of symptoms. Patients rated daytime sleepiness as the most interfering symptom; however, cataplexy, lifelike dreams (hypnagogic hallucinations), and sleep paralysis only reached the 11th, 12th, and 14th position, respectively. A recent review by Raggi et al shows that narcolepsy has an extensive impact on the health-related quality of life (HRQoL) [25]. Excessive daytime sleepiness is argued to be the symptom most affecting the HRQoL, even more than the other symptoms of the narcolepsy pentad. Excessive daytime sleepiness, however, could be viewed as a multidimensional complaint. Rather than falling asleep at inappropriate times during the day, difficulty achieving things (fourth position), lack of energy (second position), and difficulty concentrating (fifth position) can also be viewed as part of this excessive daytime sleepiness. Maski et al [17] hypothesized that subjective cognitive impairments, such as mental fog and difficulty thinking, remembering, concentrating, or paying attention, are among the most significant symptoms affecting daily life.

Narcolepsy questionnaires often primarily address the core symptoms of the disease (excessive daytime sleepiness and cataplexy) [26-29]. Recently, the Narcolepsy Severity Scale (NSS) was developed to evaluate the severity and consequences of the symptoms [30]. This 15-item questionnaire not only includes sleepiness and cataplexy but also includes hallucinations, sleep paralysis, and disturbed night time sleep. It seems to be a reliable and valid clinical tool for the quantification of narcolepsy symptoms. However, as we argue that a broader spectrum of symptoms might cause disease burden, the NSS might not be comprehensive enough to truly understand the patient with narcolepsy. Thus, despite the existence of a number of screening, diagnostic, and treatment monitoring tools, there seems to be a paucity of measures that reflect the subjective severity and the broad spectrum of narcolepsy symptoms.

Besides the experienced disease burden, the Narcolepsy Monitor also gives insight into the stability of symptoms (Table 2). Very little is known about the development of symptoms over a longer period, and patient reports might be influenced by a recall bias [31]. Day-to-day variance can not only be caused by multiple factors such as environmental changes, medication, and stress but possibly also by functional changes in the brain over time [32]. In our study, daytime sleepiness, lack of energy, and difficulty concentrating seem to be the most variable complaints, stressing the importance of a long-term monitoring tool.

### Usability

Analysis of the in-depth interviews confirmed that the patients appreciate the concept of ranking their symptoms. They experienced the use of the Narcolepsy Monitor as minimally invasive. On the other hand, the app should be improved in several aspects. Patients argued that the 3 levels did not provide enough opportunity to reflect the nuances in the change of symptoms. The 3 levels of subjective severity might also enhance the risk of a central tendency bias in which patients avoid the endpoints of a response scale and prefer responses closer to the midpoint. To keep patients motivated to use the tool over a longer period, a personal visualization of recorded data is required.

### Potential of the Narcolepsy Monitor

With the described improvements, the Narcolepsy Monitor can be used in clinical settings to monitor subjective symptom severity of narcolepsy patients and give input to clinical treatment decisions. In trails on pharmacological or nonpharmacological interventions, the app could be used as an outcome parameter. Another potential of the Narcolepsy Monitor is the further exploration as a patient-reported outcome measure (PROM). There is a growing need to gather insight into the patient perspective of a disease. Current questionnaires do not fulfill the requirements of such *patient perspective–centered* measurement. Another approach could be to make the Narcolepsy Monitor available worldwide for narcolepsy patients. This would enable the collection of large datasets to further explore, refine, and understand the narcolepsy symptom landscape.

The app was designed and built for narcolepsy patients. Narcolepsy can be seen as a *model* disease as it has a broad variety of symptoms with large differences among patients and variations over time. The concept of ranking symptoms to measure personal burden seems well suited for various kinds of disorders. Hence, by translating the app to other diseases, the principle could be more generally regarded as a *symptom monitoring tool*.

This study has some limitations. Patients were asked by their physician to participate. It is possible that the included patients are more positive toward innovations compared with those who did not choose to participate. An issue concerning the layout might have caused a bias in the results. As not all 18 symptoms were visible at first sight, patients had to actively *swipe* to see them all. This may have positively biased the ranking of the symptoms that were initially visible.

### Conclusions

This paper outlines the development and evaluation process of the Narcolepsy Monitor. Results from the pilot study suggest that this app can be used to obtain insight into the subjective severity and dynamics of narcolepsy symptoms and has interesting potential for long-term data collection and as a PROM.

## Abbreviations

HRQoL: health-related quality of life
NSS: Narcolepsy Severity Scale
PROM: patient-reported outcome measure
TU/e: Technical University Eindhoven
UCB: Union Chimique Belge
UEQ: User Experience Questionnaire
